# Assessment of myelination in infants and young children by T1 relaxation time measurements using the magnetization-prepared 2 rapid acquisition gradient echoes sequence

**DOI:** 10.1007/s00247-021-05109-5

**Published:** 2021-07-21

**Authors:** Fabienne Kühne, Wolf-Julian Neumann, Philip Hofmann, José Marques, Angela M. Kaindl, Anna Tietze

**Affiliations:** 1 Department of Pediatric Neurology, Charité – University Medicine Berlin, Berlin, Germany; 2 Movement Disorder and Neuromodulation Unit, Department of Neurology, Charité – University Medicine Berlin, Berlin, Germany; 3 Institute of Neuroradiology, Charité - University Medicine Berlin, Augustenburger Platz 1, 13353 Berlin, Germany; 4 Department of Physics and Astronomy, Interdisciplinary Nanoscience Center (iNANO), Aarhus University, Aarhus, Denmark; 5 Donders Centre for Cognitive Neuroimaging, Radboud University, Nijmegen, Netherlands

**Keywords:** Children, Infants, Magnetic resonance imaging, Magnetization-prepared 2 rapid acquisition gradient echoes, Myelination, R1 relaxometry, Reference measurements, T1 measurements

## Abstract

**Background:**

Axonal myelination is an important maturation process in the developing brain. Increasing myelin content correlates with the longitudinal relaxation rate (R1=1/T1) in magnetic resonance imaging (MRI).

**Objective:**

By using magnetization-prepared 2 rapid acquisition gradient echoes (MP2RAGE) on a 3-T MRI system, we provide R1 values and myelination rates for infants and young children.

**Materials and methods:**

Average R1 values in white and grey matter regions in 94 children without pathological MRI findings (age range: 3 months to 6 years) were measured and fitted by a saturating-exponential growth model. For comparison, R1 values of 36 children with different brain pathologies are presented. The findings were related to a qualitative evaluation using T2, magnetization-prepared rapid acquisition gradient echo (MP-RAGE) and MP2RAGE.

**Results:**

R1 changes rapidly in the first 16 months of life, then much slower thereafter. R1 is highest in pre-myelinated structures in the youngest subjects, such as the posterior limb of the internal capsule (0.74–0.76±0.04 s^−1^) and lowest for the corpus callosum (0.37–0.44±0.03 s^−1^). The myelination rate is fastest in the corpus callosum and slowest in the deep grey matter. R1 is decreased in hypo- and dysmyelination disorders. Myelin maturation is clearly visible on MP2RAGE, especially in the first year of life.

**Conclusion:**

MP2RAGE permits a quantitative R1 mapping method with an examination time of approximately 6 min. The age-dependent R1 values for children without MRI-identified brain pathologies are well described by a saturating-exponential function with time constants depending on the investigated brain region. This model can serve as a reference for this age group and to search for indications of subtle pathologies. Moreover, the MP2RAGE sequence can also be used for the qualitative assessment of myelinated structures.

**Supplementary Information:**

The online version contains supplementary material available at 10.1007/s00247-021-05109-5.

## Introduction

Myelination is a highly regulated process, primarily taking place in early childhood but continuing at a slower pace in older children and adolescents [1, 2]. It is even detectable in adults [3]. In the process, myelin sheaths are generated by oligodendrocytes in the central nervous system. They are indispensable for the proper function of neurons, axonal signaling, and synaptic plasticity.

The assessment of myelination is an integral part of any magnetic resonance imaging (MRI) examination in young children in order to detect myelination disorders and to evaluate potential secondary injuries in the brain. This is performed by qualitatively comparing predefined white matter regions of the patient’s brain with corresponding regions in normal individuals, using T1- and T2-weighted series [4]. In view of the subjectivity of this method and in order to explore the process of myelination in more detail, MRI-based approaches to quantify myelination have been proposed. Among these are magnetization transfer imaging [5], myelin water fraction imaging [6], diffusion-weighted and diffusion tensor imaging measuring fractional anisotropy, and apparent diffusion coefficient values [7, 8], as well as techniques measuring proton density, T1, and T2 relaxation times [9, 10], or simply the ratio of pixel intensity on T1- and T2-weighted images [11].

Mature white matter is characterized by an increased myelin content, which results in an increased longitudinal relaxation rate (R1) (where R1=1/T1, thus shorter relaxation time), due to the interaction of free water molecules with macromolecules [2]. Therefore, T1 mapping is a sensitive quantification method for myelination and has been investigated in previous studies of preterm babies [12], normal infants [13], children and adolescents [14]. The magnetization-prepared 2 rapid acquisition gradient echoes (MP2RAGE) sequence, which is a modification of MP-RAGE (magnetization-prepared rapid acquisition gradient echo) with two inversion times (termed TI_1_ and TI_2_) is one of the techniques that can be used for rapid and high spatial resolution mapping of quantitative relaxation map times [15, 16]. The resulting images are essentially free of radiofrequency B1 field inhomogeneity and T2* effects and are thus ideal for segmentation, tissue classification and quantification methods. Additionally, the standard weighted images obtained at each inversion time can have clinical and diagnostic value per se, even though the contrast obtained is highly dependent on the particular choice of inversion times and flip angles [17]. Once these weighted images are combined to obtain T1 maps, this parameter dependency is mostly removed, resulting in high-resolution MRI data for diagnostic purposes, largely independent of the exact parameters used at 3 tesla (T) [15].

The MP2RAGE sequence has been used to assess grey and white matter maturation in small cohorts of young children and adults showing a strong relationship between increasing R1 and advancing myelination [12, 18, 19]. These studies included either preterm infants imaged at term-equivalent or primarily older children with scant data on children under the age of 2 years, thus excluding the most relevant time for the myelination process. The aim of our study is to explore this age group in more detail, based on the following hypotheses: (1) MP2RAGE allows reliable R1 measurements in a reasonable acquisition time, (2) R1 changes in a well-defined temporal and spatial pattern in white and grey matter, (3) R1 can be altered in diseases affecting white matter and (4) R1 mapping can provide useful diagnostic information that cannot be obtained by qualitative assessment alone. We want to draw attention to the power of quantitative imaging in clinical practice that can be achieved by using a diagnostic sequence without adding extra scan time. We illustrate this by presenting R1s of children without pathologies discernible in MRI and of patients with different pathologies supposedly affecting white matter.

## Materials and methods

### Subjects

The subjects for this study were identified by a board-certified neuroradiologist (A.T., with 9 years of experience in general neuroradiology, 4 years exclusively in pediatric neuroradiology). The picture archiving and communication system (PACS) was searched for examinations including the MP2RAGE sequence from July 2017 (the time at which MP2RAGE was introduced as a standard sequence at our department) to April 2020. The only search criterion was MP2RAGE. With the aim of comparing our results to the available studies of older children, we included children up to the age of 6 years.

The result of the search was then divided into two subgroups by evaluating previous or subsequent clinical history documented in the electronic medical record system of our hospital as well as all previous or subsequent imaging findings. The first group included cerebral low-risk (termed MRI-negative) children examined for tics, suspected or first seizure, absence seizures, suspected benign enlargement of the subarachnoid spaces or hydrocephalus, cleft palate, scalp malformations, suspected acute infections, trauma, headaches, breath-holding spells, or developmental delay (indications for MRI examinations, see Table 1). These children did not show any MRI pathology, not even subtle white matter signal abnormalities on any sequence acquired as part of our routine imaging protocol (including MP2RAGE). The second, MRI-positive group consisted of patients who were diagnosed with various pathologies affecting the white matter. We excluded 28 patients with extensive white matter loss or severely motion- or artifact-degraded series.

**Table 1:** Frequencies of abnormalities in the 33 MRI-positive patients, and MRI indications for MRI-negative subjects^a^

Abnormality	Number of MRI-positive patients	Number of MRI-negative patients
Focal cortical dysplasia, types IIa and IIb	3	0
Heterotopias	7	0
Polymicrogyria	2	0
Pachygyria	3	0
Tuberous sclerosis complex	2	0
Hypo- and dysmyelination	3	0
Periventricular gliosis	6	0
Atrophy	12	0
Corpus callosum hypoplasia	4	0
Microbleeds/periventricular calcifications	5	0
Encephalitis	2	0
Suspected seizure or first seizure, including absence seizures and tics	0	62
Suspected benign enlargement of the subarachnoid spaces or hydrocephalus	0	7
Cleft palate	0	1
Developmental delay	0	6
Scalp malformations	0	4
Suspected acute infections	0	2
Breath-holding spells	0	2
Headaches	0	10
Trauma	0	1

^a^Note that patients can be affected by several pathologies

This retrospective study is part of a different study in children with brain tumors, where data for comparison are needed and as such approved by the local ethics committee. Informed consent was waived due to the retrospective nature of this study.

### MRI acquisition

MRI was performed on a Skyra 3-T system (Siemens Healthineers, Erlangen, Germany) with a 64-element or an 8-element neonatal head coil (babies <2 months). The MP2RAGE sequence took 5 min, 47 s (sagittal, voxel size 1×1×1 mm^3^, field of view 256 mm^2^, repetition time [TR]=5,000 ms, echo time [TE]=2.98 ms, TI_1_=700 ms, TI_2_=2,500 ms, flip angle_1_=4°, flip angle_2_=5°, 7.1 ms echo spacing, 176 slices, generalized autocalibrating partial parallel acquisition [GRAPPA] acceleration factor 3). The MRI protocol also included an axial, two-dimensional (2-D), fat-saturated T2 turbo spin echo (TSE) sequence (voxel size 0.4×0.4×3 mm^3^; field of view 230 mm^2^; TR=5,000 ms; TE=100 ms; flip angle=150°; GRAPPA acceleration factor 2; duration 2 min, 12 s) and sagittal MP-RAGE (voxel size 0.9×0.9×0.9 mm^3^, field of view 240 mm^2^, TR=2,300 ms, TE=2.32 ms, TI=900 ms, flip angle=8°, GRAPPA acceleration factor 2) as well as 2-D or three-dimensional (3-D) T2 fluid-attenuated inversion recovery (FLAIR), T2*, and diffusion-weighted imaging (DWI) sequences. Contrast agent was rarely administered for the MP-RAGE, but never for the MP2RAGE. MRI was usually performed under sedation (50–100 mg/kg chloralhydrate perorally) in children <4 years old and in general anesthesia (combined intravenous propofol and isoflurane per inhalation) for older children if necessary.

### MRI data analysis

**Fig. 1:**
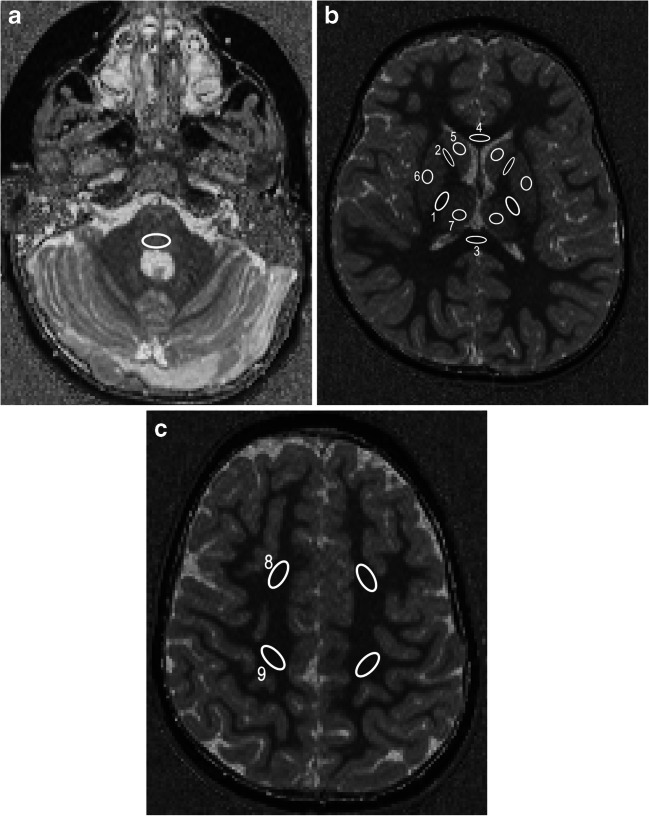
Axial T1 maps in a 2-year-old girl. **a–c** T1 values were measured in the dorsal pons (**a**); the posterior limb of the internal capsule (*1*), the anterior limb of the internal capsule (*2*), the splenium (*3*) and genu (*4*) of the corpus callosum, the caudate nucleus (*5*), the putamen (*6*), and the thalamus (*7*) (**b**); and the frontal (*8*) and central (*9*) white matter of the centrum semiovale (**c**). For illustration purposes, the regions are not drawn using the paintbrush tool in ITK-SNAP

The bias-field corrected MP2RAGE data were converted to NIfTI (Neuroimaging Informatics Technology Initiative) format and T1 maps were generated using MatLab 9.5 (Mathworks, Natick, MA) [15]. The T1 map generation step is computationally inexpensive and takes a few seconds on a standard PC. The T1 maps were then loaded into the image viewer ITK-SNAP [20]. The following regions of interest (ROIs) were defined manually using the paintbrush tool: bilateral posterior limb of the internal capsule (PLIC), anterior limb of the internal capsule (ALIC), central white matter and frontal white matter in the centrum semiovale, the genu and splenium of the corpus callosum, and the posterior pons. Moreover, grey matter ROIs were defined in the putamen, the caudate nucleus, and the thalamus/pulvinar (Fig. 1). This task was performed by a medical student (F.K.) blinded to clinical information. The ROI sizes within one region were kept as stable as possible between subjects, with some degree of constraint imposed by the anatomical structure. Mean T1 values (±standard deviation [SD]) were extracted for each ROI and subject.

In order to assess inter- and intra-rater reliability, a subset of 30 MRI-negative subjects was reanalyzed (for bilateral structures, the left hemisphere only) by the same medical student and the board-certified neuroradiologist. We included subjects with the same age distribution by dividing the entire cohort into 12 bins per 6 months of age and randomly choosing the same number of subjects from each bin.

Finally, we assessed whether the additional examination time of almost 6 min for the MP2RAGE sequence can be diagnostically justified, even in the absence of a quantitative analysis. To this end, we qualitatively described the myelination progression as represented on the MP2RAGE sequence compared to a standard T2-weighted sequence and MP-RAGE. The scanner routinely also generates the TI_1_ and TI_2_ data from the MP2RAGE acquisition and both were included in this evaluation. Images were compared to standard T1- and T2-weighted images by the experienced neuroradiologist with regard to myelinated/unmyelinated white matter and white/grey matter and whether the MP2RAGE signal temporally and spatially followed the established patterns on conventional T1- and T2-weighted images [4].

### Statistical analysis

R1 for the MRI-negative subjects was plotted for different brain regions as a function of age using Igor Pro (WaveMetrics, Lake Oswego, OR). A saturating-exponential model was fitted to the data according to

$$ R1=R{1}_{\infty }+A{e}^{-t/\tau } $$

where *A* is a (negative) constant, *t* is the age and τ is the time constant for the myelination process. In this description, R1_∞_ is the R1 value at infinite age and R1_∞_+A is the R1 value at birth. The fit is performed by minimizing the sum of the squared distances between data points and the model (least-square fit), taking into account the individual uncertainties of the data points.

The key parameter resulting from this analysis is the time constant 𝜏 and its uncertainty. The same analysis was performed individually for male and female subjects. The R1(*t*) values for the MRI-positive group were plotted along with the data for the MRI-negative subjects.

For the assessment of the inter-/intra-rater reliability, the intraclass correlation coefficient (ICC) was calculated using the ‘irr’ package in R (R Foundation for Statistical Computing, Vienna, Austria). Bland-Altman plots were generated using the ‘ggplots2’ and ‘BlandAltmanLeh’ packages in R.

## Results

### Subjects

Ninety-four MRI-negative children (60 males, 34 females) and 36 MRI-positive children (24 males, 12 females) were included (Table 1). Five MRI-negative and four MRI-positive children were born prematurely and ages were corrected accordingly. The mean age of the MRI-negative group was 34.4 months (SD 20.9 months, range: 3 months-6 years; 38 subjects ≤24 months+2 days). The mean age of the MRI-negative group was 39.7 months (SD 22.1 months, range: 3.5 months to 6 years; 11 subjects ≤24 months+2 weeks). The mean age of the subset used for the inter-rater reliability assessment was 34.5 months (SD 21.1 months, range: 4 months to 6 years; 13 subjects ≤24 months+5 weeks).

### R1 measurements in white and grey matter ROIs

**Fig. 2:**
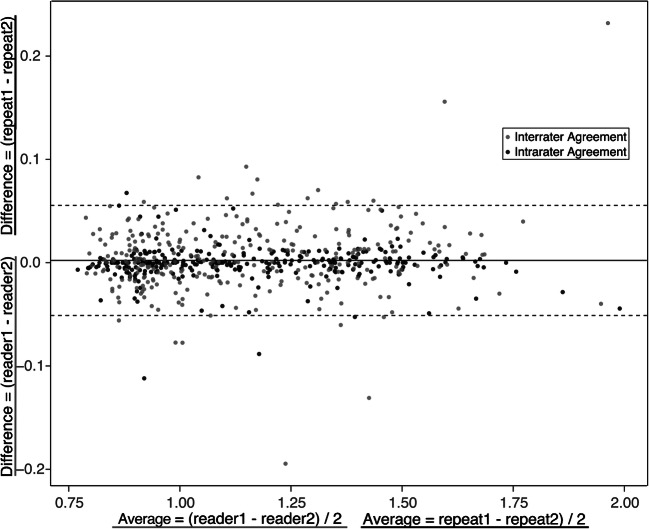
A Bland-Altman plot shows unbiased agreement for all regions (for bilateral regions, only the left side is measured) for the inter-rater (*black dots*) and the intrarater (*grey dots*) correlation in a subset of 30 MRI-negative subjects

The ROIs could be easily outlined in all MRI-negative subjects, whereas, in the MRI-positive group, the ROI size had to be adjusted in some patients due to atrophy or could not be defined at all if a structure was not present (e.g., the splenium in the case of corpus callosum hypoplasia). The average number of voxels for ROIs ranged between 13.94 (±2.04) for small structures such as the ALIC in MRI-positive subjects and 65.16 (±1.99) in larger structures such as the splenium in MRI-negative subjects. Details are reported in Online Supplementary Material 1. The ICC of the inter-rater reliability was on average 0.970 (SD 0.02; minimum for the posterior pons: 0.946 and maximum for the frontal white matter: 0.99). For the intra-rater reliability, the ICC was on average 0.992 (SD 0.01; minimum for the thalamus: 0.977 and maximum for the frontal white matter: 0.99). A Bland-Altman plot showed good agreement and no bias was found (Fig. 2).

**Fig. 3:**
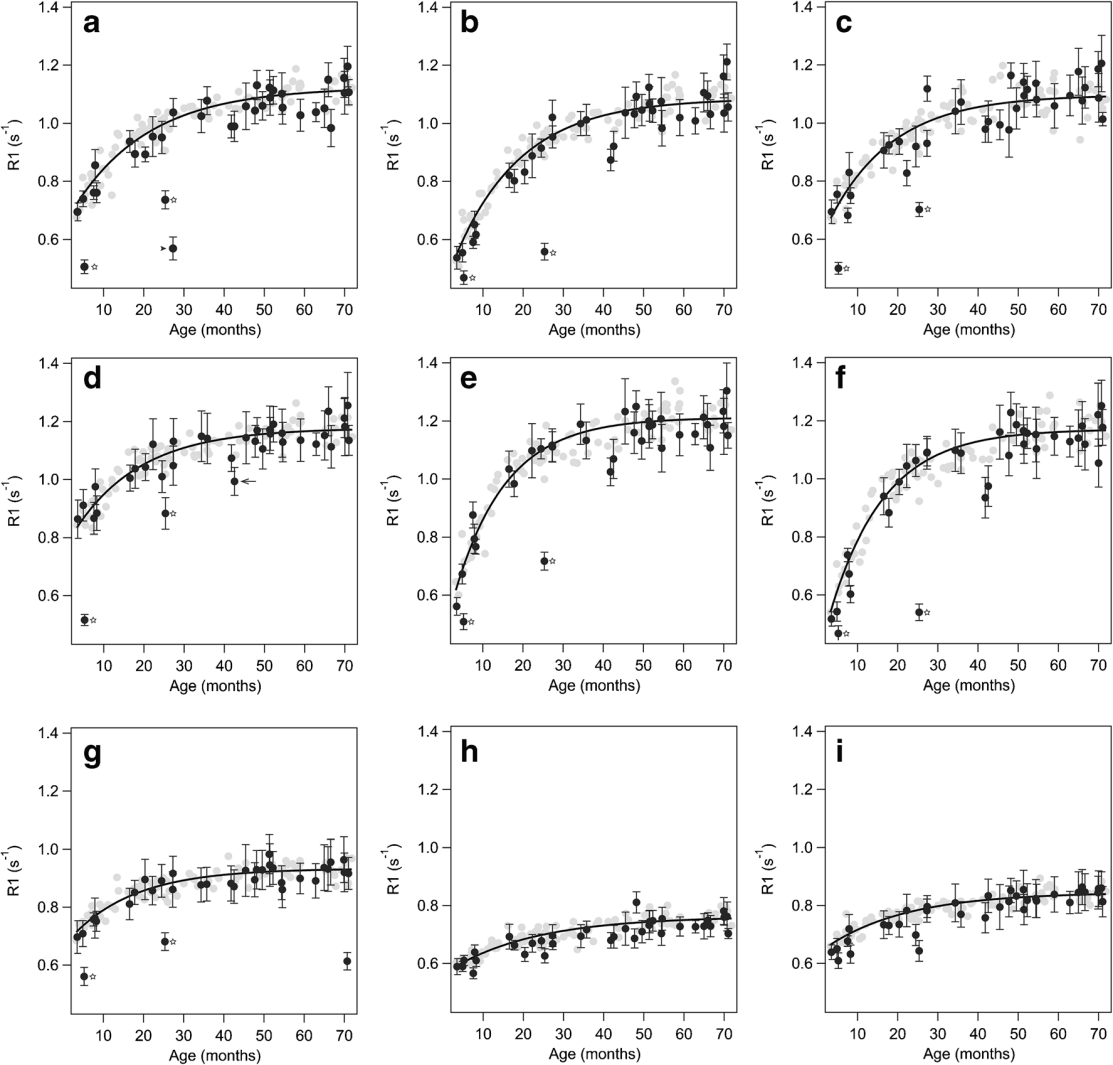
Average relaxation rate (R1) values for MRI-negative patients (*grey dots*) and for patients with different pathologies (*black dots*, including the standard deviations as error bars). The *solid black lines* are the best fit of a saturating-exponential model to the grey data points. For bilateral structures, only the right side is shown (additional data is in Online Supplementary Material 3). The myelination rate is given as the time constant τ ± uncertainty. **a** Central white matter (WM), τ =18.7±2.2 months. **b** Frontal WM, τ =16.5±1.2 months. **c** Anterior limb of the internal capsule, τ =15.8±1.9 months. **d** Posterior limb of the internal capsule, τ =16.5±3.1 months. **e** Splenium of the corpus callosum, τ =12.9±1.1 months. **f** Genu of the corpus callosum, τ =13.6±1.0 months. **g** Posterior pons, τ =15.9±3.8 months. **h** Putamen, τ =21.5±4.0 months. **i** Thalamus, τ =20.1±5.3 months. Two patients have particularly low R1 in white matter and are marked by *stars* (**a–g**). A younger boy (5.2 months old) has a 18q deletion syndrome, the older boy (25.3 months old) has Pelizaeus-Merzbacher disease. A 27.3-month-old boy (*arrowhead* in **a**) has low R1 due to dysmyelination after a congenital cytomegalovirus infection. A 42.5-month-old boy (*arrow* in **d**) with brain atrophy and nonspecified epilepsy has relatively low R1 in the posterior limb of the internal capsule, which is not recognizable on conventional series (see Fig. 4)

Plots of R1 as a function of age in different ROIs are shown in Fig. 3, along with myelination rates for each region. Regions with low τ achieve maturation fast as opposed to regions with high τ, where myelin matures slowly. The maturation in the corpus callosum is faster than that in the frontal/central white matter or in the deep grey matter. In a sub-analysis, we addressed potential gender differences regarding myelination rates. The values of male and female subjects agree within their respective uncertainties except for the corpus callosum and right ALIC, where the uncertainties do not overlap, but only just, thus not suggesting any relevant differences. Expected R1 value as a measure for myelination state for all MRI-negative individuals as calculated from the best-fit model at birth, at 12 months, and at 24 months are given in Table 2. R1±SD for each ROI in all MRI-negative subjects are given as Online Supplementary Material 2.

**Table 2:** Relaxation rate (R1) values for each region in MRI-negative children at birth, 12 months and 24 months, calculated from the best-fit models in Fig. 3 and Online Supplementary Material 2

Region of interest	R1 at birth, right side (s^−1^)	R1 at birth, left side (s^−1^)	R1 at 12 months, right side (s^−1^)	R1 at 12 months, left side (s^−1^)	R1 at 24 months, right side (s^−1^)	R1 at 24 months, left side (s^−1^)
Posterior limb of the internal capsule	0.76	0.74	0.97	0.97	1.08	1.08
Anterior limb of the internal capsule	0.57	0.58	0.85	0.86	0.98	0.99
Central white matter	0.64	0.63	0.87	0.87	0.99	0.99
Frontal white matter	0.43	0.43	0.77	0.76	0.93	0.93
Genu of the corpus callosum	0.37		0.84		1.03	
Splenium of the corpus callosum	0.44		0.91		1.09	
Posterior pons	0.66		0.81		0.87	
Thalamus	0.63	0.64	0.73	0.73	0.78	0.78
Putamen	0.56	0.57	0.65	0.65	0.70	0.69
Caudate nucleus	0.57	0.56	0.63	0.63	0.66	0.66

The standard deviation for all values is ≤0.01 s^−1^

**Fig. 4:**
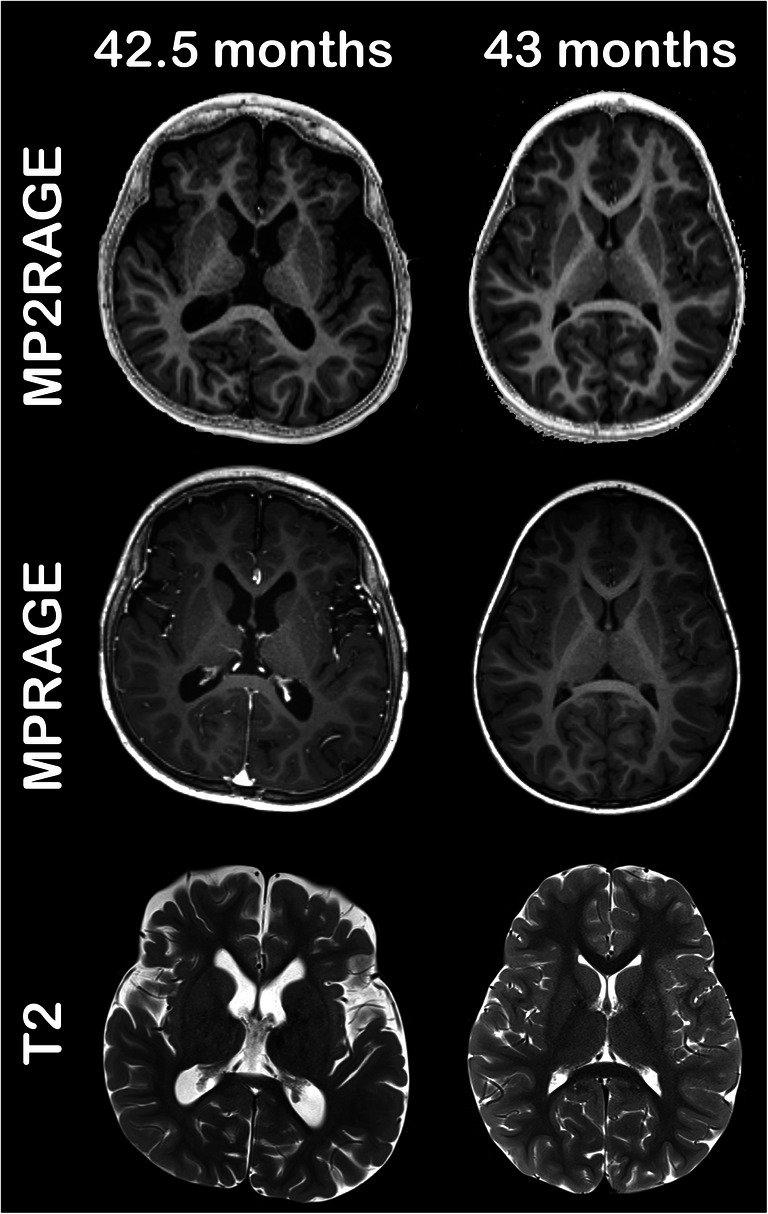
A 42.5-month-old boy with brain atrophy and unspecified epilepsy. Axial MP2RAGE (magnetization-prepared 2 rapid acquisition gradient echoes), contrast-enhanced MP-RAGE (magnetization-prepared rapid acquisition gradient echo) and T2-weighted images show very similar signal intensities in the posterior limb of the internal capsule as in an MRI-negative peer (a 43-month-old girl; her MP-RAGE image is not contrast enhanced). Note the different relaxation rate (R1) values in the posterior limb of the internal capsule in Fig. 3, which cannot be appreciated qualitatively on conventional sequences (right: 0.99±0.05 s^−1^, left: 1.00±0.05 s^−1^ for the boy; right: 1.08±0.04 s^−1^, left: 1.07±0.04 s^−1^ for the MRI-negative girl)

The R1 values of the subjects in the MRI-positive group are mostly consistent with the model derived from the MRI-negative subjects in that the best-fit model lies within the uncertainties of the corresponding data points. This means that the individuals in question show appropriate myelination and no measurable white matter disease. Two patients, marked with stars in Fig. 3, showed lower R1 values in all white matter ROIs; both patients were severely hypomyelinated. The younger patient (5.2 months old) had been diagnosed with a 18q deletion syndrome and the older one (25.3 months old) with Pelizaeus-Merzbacher disease. The 27.3-month-old child had extensive lesions in the central white matter due to a congenital cytomegalovirus infection (Fig. 3). The 70.7-month-old child (5.9 years old) showed low R1 in the posterior pons due to signal abnormalities as a result of rhombencephalitis. While these pathologies are usually obvious on conventional MRI sequences, subtle R1 changes due to dys- or hypomyelination can be more difficult to recognize. This is illustrated in Fig. 4, where the bias field corrected MP2RAGE, conventional MP-RAGE (contrast enhanced for the MRI-positive subject) and T2 TSE images are shown in a 42.5-month-old boy (3.5 years old) with global brain atrophy and non-specified epilepsy, and a 42.9-month-old (3.6 years old) MRI-negative peer. There are no clear differences in the conventional images, but the R1 values in the PLIC differ between MRI-positive and MRI-negative children (Figs. 3 and 4).

**Fig. 5:**
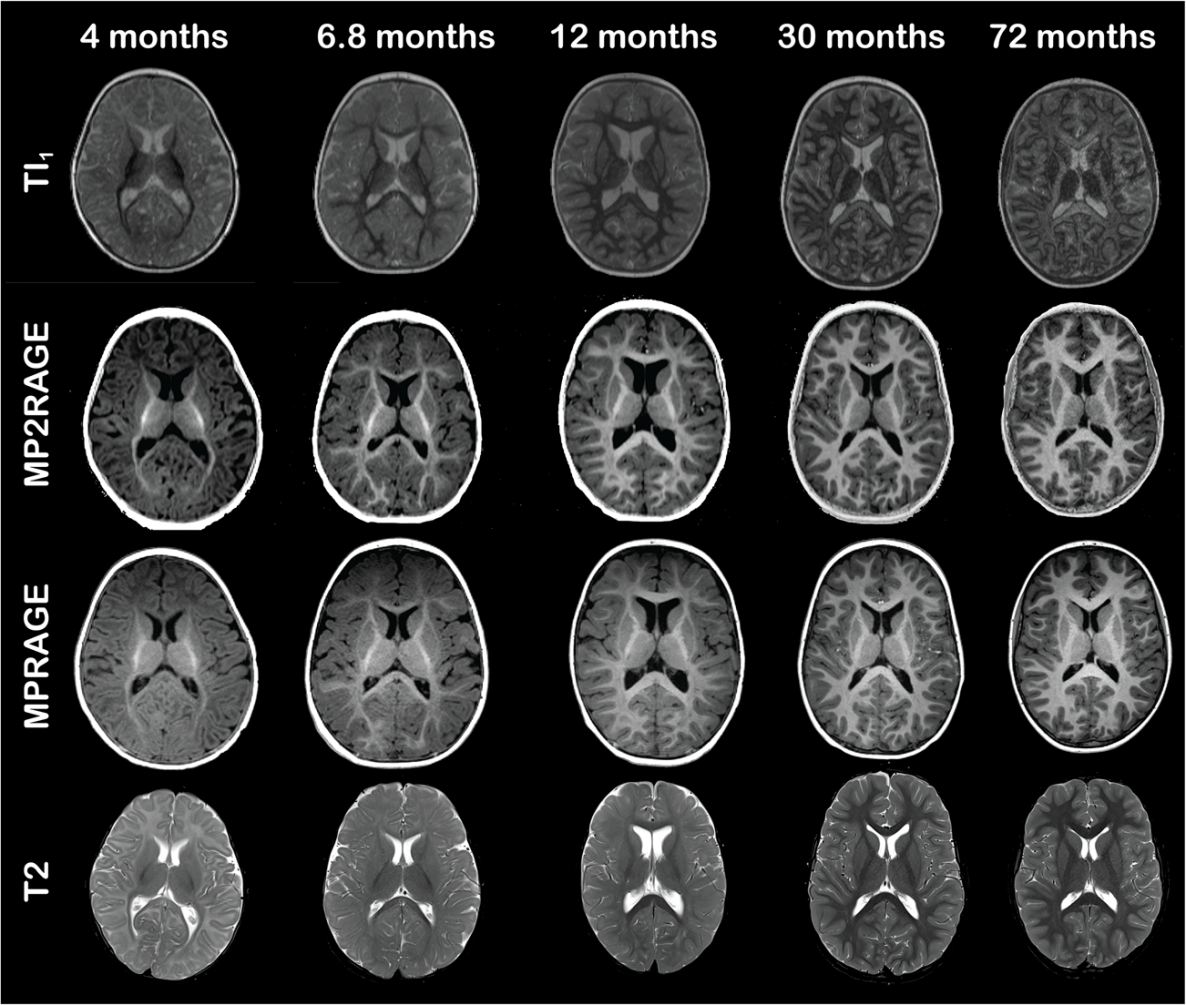
Axial TI_1_, MP2RAGE (magnetization-prepared 2 rapid acquisition gradient echoes), MP-RAGE (magnetization-prepared rapid acquisition gradient echo) and T2-weighted images at the level of the posterior limb of the internal capsule for five MRI-negative children (boys ages 4 months, 6.8 months and 30 months, and girls ages 12 months and 72 months)

In a qualitative evaluation, the myelinated structures are easily recognized on MP2RAGE and especially on TI_1_ at a young age (<12 months) due to the excellent contrast between myelinated and unmyelinated white matter, as well as between grey and white matter. Examples for MRI-negative subjects (4 months, 6.8 months, 12 months, 30 months and 72 months) are given in Fig. 5; the TI_1_ images of the MP2RAGE, bias-field-corrected MP2RAGE, conventional MP-RAGE and T2 TSE are shown (for the same individuals at the centrum semiovale, see Online Supplementary Material 2). Early myelination under the age of 12 months is well discernible on the TI_1_ (hypointense) and MP2RAGE (hyperintense) as demonstrated in Fig. 5 and Online Supplementary Material 4. Myelin maturation follows the established spatiotemporal pattern and shows an increasing hyperintensity on MP2RAGE with excellent contrast between white and grey matter at an older age. The TI_1_ images appear to be especially useful for ages <12 months, when the striking hypointensity of early myelination slowly changes to isointensity for mature myelin, again following the well-known spatiotemporal sequence. Early myelin is TI_1_-hypointense and becomes TI_1_-isointense at a later stage (Fig. 5). This results in signal inversion when comparing the 4-month-old infant to the 12-month-old child (Fig. 5). In contrast to conventional MP-RAGE and T2 TSE data where signal changes are very subtle >24 months, the TI_1_ signal first reaches an adult pattern around the age of 6 years. The TI_2_ images did not contribute additional anatomical information compared to conventional MP-RAGE data and are not shown in the figure.

## Discussion

We measured R1 values in the white and deep grey matter of MRI-negative children using a high-resolution MP2RAGE sequence with the aim of describing the change in R1 in young children as a result of increasing myelination, and evaluating a quantitative R1 mapping method that is easily applicable in the clinical practice. We were particularly interested in children <24 months old, but included children between 2 years old and 6 years old in order to compare our results with previous studies [10, 18]. The R1 values of the MRI-negative children were further compared to those of patients with different pathologies affecting the white matter.

T1 mapping in children has been performed in preterm newborns at term-equivalent, as well as in children and adolescents, but few data are available for children between term and 24 months old, when the bulk of myelination takes place [10, 12, 18, 19]. We showed a rapid increase of R1 in white matter in almost all predefined ROIs during this period, leveling off between 2 years of age and 6 years of age. Areas with densely packed axons, like the corpus callosum, showed the greatest R1 increase, starting with low values of 0.37±0.01 s^−1^ (genu of the corpus callosum) and 0.44±0.01 s^−1^ (splenium) when myelination is not yet initiated (note that the quoted values and uncertainties refer to the result of the best-fit model given in Table 2). By contrast, the R1 increase in white matter was lowest in the PLIC where early myelination stages are present at birth with correspondingly higher R1 (0.76±0.01 s^−1^ and 0.74±0.01 s^−1^ for the right and left side, respectively). The pontine tegmentum (posterior pons) is another early myelinated structure, with an R1 value of 0.66±0.01 s^−1^ at birth and little increase over time, never exceeding 1.0 s^−1^. This might be caused by intermingled grey matter structures, such as the nuclei of the 5th–8th cranial nerves. We found that the myelination rate in the corpus callosum is faster than that in the centrum semiovale. As expected, the deep grey matter shows little change. In general, the anatomical distribution and temporal evolution of R1 in our population corresponds well with the expected pattern in infants and young children [10, 21, 22]. The R1 values are also comparable to those measured with the same technique in the 15 children <5 years old by Eminian et al. [18], but that study included only 5 children under the age of 2 years. Our values also appear to be consistent with a study of preterm infants scanned at term-equivalent [12] when extrapolating the results of that study to an older age.

The post-processing of MP2RAGE data to produce T1 maps is computationally inexpensive and took only seconds on a standard PC. It would be helpful to calculate the T1 maps directly on the scanner console to make them available in line with other diagnostic results, in the same way as diffusion-weighted images. Ideally, the results could be displayed as difference maps between the individual patient data and an age-matched template originating from a large set of normal data. This would directly highlight the T1 value discrepancies on a voxel-wise basis.

The quantitative nature of T1 mapping directly lends itself to longitudinal and cross-center comparisons, making the approach superior to semiquantitative methods as, for example, recently described by Flood et al. [23], where T1 signal intensities were normalized to cerebrospinal fluid. The most important shortcoming of such approaches is the lack of bias field corrections that inevitably leads to inaccurate measurements. Moreover, such intensity ratios cannot be compared across different MRI systems and different radiofrequency coils.

We calculated R1 for patients with disorders of neuronal proliferation, migration or organization as well as for patients with atrophy as a result of different underlying pathologies. Not surprisingly, myelination is very similar in MRI-negative patients with many of these pathologies, but is altered in gliotic tissue or in hypomyelination disorders. It is conceivable that R1 or difference maps can alert the less experienced radiologist to subtle and symmetrically distributed pathologies. Moreover, some pathologies might not be captured by the eye at all, but could be detected on quantitative maps (Fig. 3). This would help in diagnostic contexts but could also serve as an important tool for research purposes, especially if longitudinal, inter-subject and cross-center data are to be compared.

Quantitative relaxometry allows a detailed and objective tissue characterization reflecting physiology and pathophysiology by providing absolute biophysical parameters on a voxel-wise level. T1 mapping is largely independent of the parameter setting in the MP2RAGE sequence. While this undoubtedly represents an advance in imaging diagnostics, quantitative imaging will always be an additional tool to the qualitative evaluation of standard weighted images in the clinical context. Myelin maturation has been described extensively on T1- and T2-weighted images ([4] serves as just one example), but to the best of our knowledge, MP2RAGE image contrasts have so far not been described. Therefore, we have compared the contrast between myelinated and unmyelinated white matter as well as the grey/white matter contrast in MP2RAGE images (including TI_1_ and TI_2_ used for bias field correction) to that in conventional sequences. This was done on a descriptive basis and not as the primary focus of this study. We found that TI_1_ images, especially, are very useful for the visual assessment of myelination in infants <12 months old due to the sharp contrast between myelinated and unmyelinated white matter. The reason for this is that early myelination is very hypointense, i.e. the signal is nulled by the inversion pulse and contrasts excellently with unmyelinated white matter as well as with grey matter. As myelination proceeds, the signal reverts on TI_1_ because more mature myelin is no longer suppressed and becomes isointense to cortex. This sequence of first hypointensity and then isointensity is not observed in T1- and T2-weighted images, but the spatial order of the changes on T1- and T2-weighted images follows the well-established pattern. Moreover, the observed myelination process in TI_1_ images extends over a much longer period of time than in the corresponding conventional sequences. Indeed, while few changes are seen after the age of 24 months on conventional T1- and T2-weighted images, the adult pattern on TI_1_ appears first to be reached around the age of 6 years. We can only speculate as to the causes of the signal inversion and time dependence of the TI_1_ images. It is likely that these mirror the development described by Barkovich [2]; myelin formation starts with primary transitional membranes formed by oligodendrocytes, followed by a highly regulated maturation process, in which the myelin composition is constantly changing before it is becoming more and more compacted [24]. These changes continue until adulthood and have been tracked by other techniques, such as diffusion tensor or kurtosis imaging [7, 25–27]. In a clinical context, we also use the TI_1_ to find subtle pathologies in children >24 months old, such as focal cortical dysplasias or gliosis, where the myelin composition is altered, causing signal suppression again and making these changes very obvious on this series. It has, however, to be kept in mind that the detailed signal contrasts of TI_1_ and TI_2_ are highly dependent on parameter settings, in particular inversion times and flip angles, as is the case for other standard sequences as well.

There are some important limitations of our study. First of all, in this retrospective study we did not include entirely healthy individuals due to ethical reservations. We selected patients primarily referred for suspected first-time seizures, headaches, trauma, etc. If no morphological pathology was found, the corresponding individuals were included in the MRI-negative group. Theoretically, minor pathologies might be present and cause less accurate measurements. We included 38 children <24 months old, which is not a large number, but is considerably higher than in a comparable existing study [18]. The myelination time constants, an important result of our study, might change when increasing the number of individuals, but we do not expect substantial changes. An indication for the stability of the τ values is the fact that similar results are obtained for the subsets of male and female patients. A further limitation of this study is that data were acquired under clinical conditions, where quality has to be balanced between available scanning time in a busy imaging department, the need for short sedation, and general anesthesia time. To account for these constraints, the MP2RAGE sequence provided by the vendor was adapted by decreasing the phase resolution from 100% to 80%. This leads inevitably to increased noise. On the other hand, the signal-to-noise ratio is possibly improved by the high-quality 64-channel head coil used in this study. A source of systematic bias could be B1 field inhomogeneities. It should be noted, however, that such effects are small on 3-T scanners (variations of ±10% are typically expected in the brain) and should be even smaller when imaging young children with small heads. When analyzing our specific protocol, the bias introduced by a 10% change in transmitted radiofrequency power resulted in 2.5% and 2.7% changes of grey and white matter R1 values, respectively, while the temporal changes in R1 observed in our study varied between 14% (right putamen) and 50% (splenium). The minor significance of field homogeneities further allows us to conclude that our data can be directly compared to other maturation studies using MP2RAGE [18]. Although MP2RAGE is only able to remove received field biases, while transmit fields are only partially accounted for [16], it has recently been shown that MP2RAGE has a high reproducibility and provides precise T1 maps even at 7 T, where a higher B1 field inhomogeneity has to be accounted for [28]. Finally, the qualitative description of image contrasts during different myelination steps using MP2RAGE is subjective in nature; however, this part of the study is merely intended to draw attention to the additional diagnostic benefit of the sequence.

## Conclusion

We have provided reference data for myelination rates in different regions of the brain. We have also presented data from children with different pathologies and found that myelination in these children is very similar to that of MRI-negative individuals in most patients, apart from those with global hypomyelination conditions. In addition, we stress the diagnostic benefit of the TI_1_ images that not only enable the easy identification of early myelination stages, but also appear to show later myelination steps.

## Supplementary Information

Online Supplementary Material 1 (PDF 134 kb)

**Online Supplementary Material 2** Data points: average R1 values for magnetic resonance imaging (MRI)-negative individuals with standard deviations represented as bars. The black line is the best fit saturating-exponential function. The myelination rate is given as the time constant τ ± uncertainty. Central white matter (WM) (**a**) right, τ=18.7±2.2 months; (**b**) central WM left, τ=17.5±2.1 months; (**c**) frontal WM right, τ=16.5±1.2 months; (**d**) frontal WM left; τ=17.5±2.1 months; (**e**) anterior limb of the internal capsule right, τ=15.8±1.9 months; (**f**) anterior limb of the internal capsule left, τ=16.4±2.3 months; (**g**) posterior limb of the internal capsule right, τ=16.5±3.1 months; (**h**) posterior limb of the internal capsule left, τ=15.2±2.6 months; (**i**) splenium of the corpus callosum, τ=12.9±1.1 months; (**j**) genu of the corpus callosum, τ=13.6±1.0 months; (**k**) posterior pons, τ=15.9±3.8 months; (**l**) putamen right, τ=21.5±4.0 months; (**m**) putamen right, τ=225.8±5.5 months; (**n**) caudate nucleus right, τ=21.5±6.5 months; (**o**) caudate nucleus left:, τ=20.5±6.1 months; (**p**) thalamus right, τ=20.5±5.3 months, and (**q**) thalamus right, τ=20.6±5.6 months (PDF 795 kb)

Online Supplementary Material 3 (PNG 807 kb)

Online Supplementary Material 4 Axial TI_1_, MP2RAGE, MP-RAGE and T2-weighted images at the level of the centrum semiovale in different age groups (boys ages 4 months, 6.8 months and 30 months, and girls ages 12 months and 72 months)(PNG 9.19 mb)
