# Supplementary material for: Assessment of myelination in infants and young children by T1 relaxation time measurements using the magnetization-prepared 2 rapid acquisition gradient echoes sequence
Source: Pediatr Radiol. 2021 Jul 21;51(11):2058–68. doi: 10.1007/s00247-021-05109-5 (PMC8476383; doi:10.1007/s00247-021-05109-5)
Supplement: Supplementary file 1 — (PDF 134 kb) [file 247_2021_5109_MOESM1_ESM.pdf]

Online Supplementary Material 1 Average number of voxels with standard deviations (SD) in white and grey matter regions of interest for MRI-negative and MRI-positive subjects

Region of interest (ROI)	Average # voxels (±SD) in MRI-negative subjects		Average # voxels (±SD) in MRI-positive subjects	
	Right side	Left side	Right side	Left side
Posterior limb of the internal capsule	38 (0)	38 (0)	33.14 (7.42)	33.14 (7.42)
Anterior limb of the interior capsule	15.99 (0.24)	15.98 (0.15)	13.97 (2.10)	13.94 (2.04)
Central white matter	44 (0)	44.12 (0.87)	41 (7.92)	40.97 (7.99)
Frontal white matter	44 (0)	44 (0)	44 (0)	44 (0)
Genu of corpus callosum	58.6 (1.88)		49.82 (14.37)	
Splenium of the corpus callosum	65.16 (1.99)		53.18 (17.07)	
Posterior pons	24.01 (0.11)		23.56 (1.58)	
Putamen	21 (0)	21 (0)	21 (0)	21 (0)
Caudate nucleus	50 (0)	50 (0)	50 (0)	50 (0)
Thalamus	44 (0)	44 (0)	44 (0)	44 (0)
