# Supplementary figures and images for: Assessment of myelination in infants and young children by T1 relaxation time measurements using the magnetization-prepared 2 rapid acquisition gradient echoes sequence
Source: Pediatr Radiol. 2021 Jul 21;51(11):2058–68. doi: 10.1007/s00247-021-05109-5 (PMC8476383; doi:10.1007/s00247-021-05109-5)

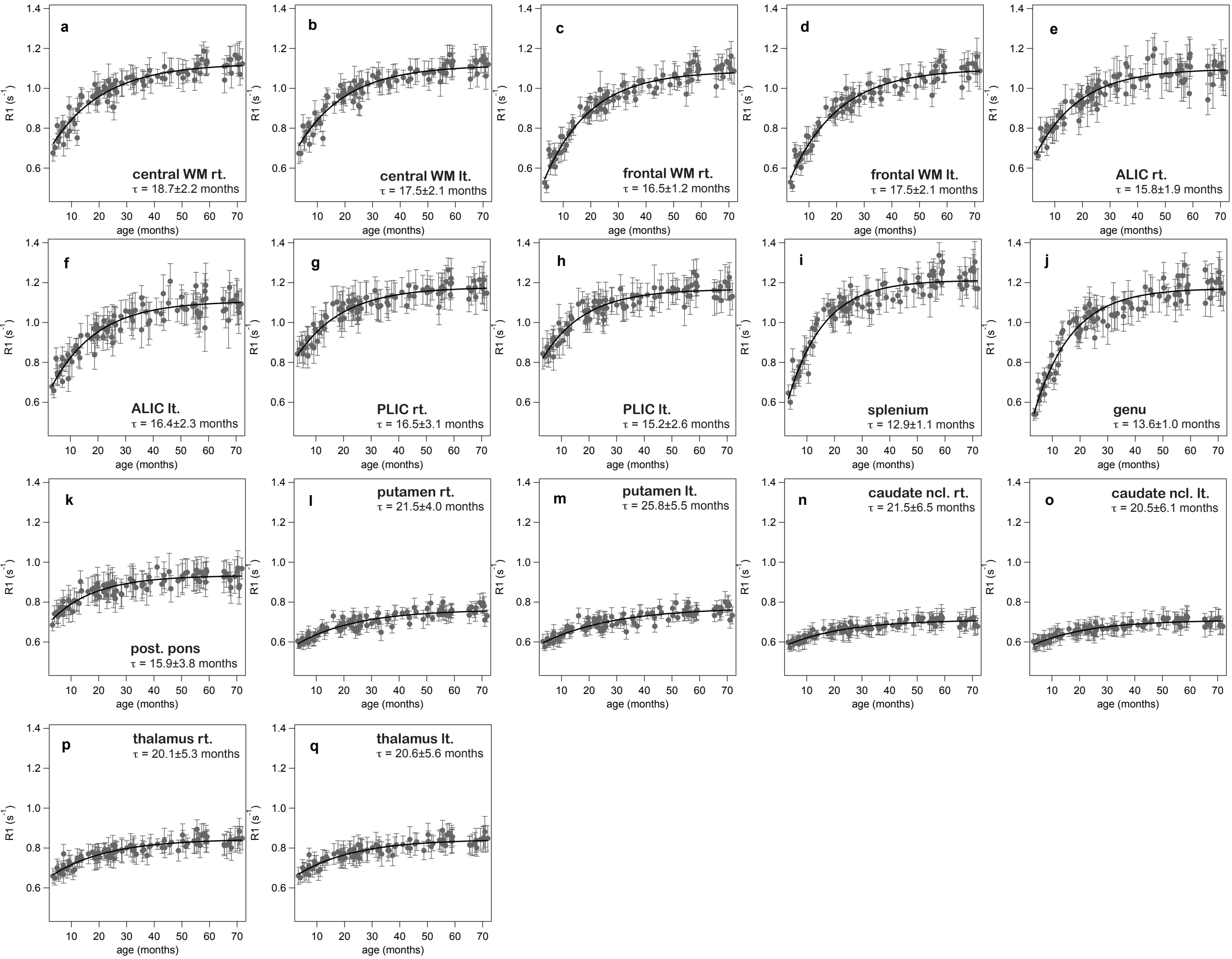

Supplement: Supplementary file 2 — Online Supplementary Material 2 Data points: average R1 values for magnetic resonance imaging (MRI)-negative individuals with standard deviations represented as bars. The black line is the best fit saturating-exponential function. The myelination rate is given as the time constant τ ± uncertainty. Central white matter (WM) (a) right, τ=18.7±2.2 months; (b) central WM left, τ=17.5±2.1 months; (c) frontal WM right, τ=16.5±1.2 months; (d) frontal WM left; τ=17.5±2.1 months; (e) anterior limb of the internal capsule right, τ=15.8±1.9 months; (f) anterior limb of the internal capsule left, τ=16.4±2.3 months; (g) posterior limb of the internal capsule right, τ=16.5±3.1 months; (h) posterior limb of the internal capsule left, τ=15.2±2.6 months; (i) splenium of the corpus callosum, τ=12.9±1.1 months; (j) genu of the corpus callosum, τ=13.6±1.0 months; (k) posterior pons, τ=15.9±3.8 months; (l) putamen right, τ=21.5±4.0 months; (m) putamen right, τ=225.8±5.5 months; (n) caudate nucleus right, τ=21.5±6.5 months; (o) caudate nucleus left:, τ=20.5±6.1 months; (p) thalamus right, τ=20.5±5.3 months, and (q) thalamus right, τ=20.6±5.6 months (PDF 795 kb) [file 247_2021_5109_MOESM2_ESM.pdf]

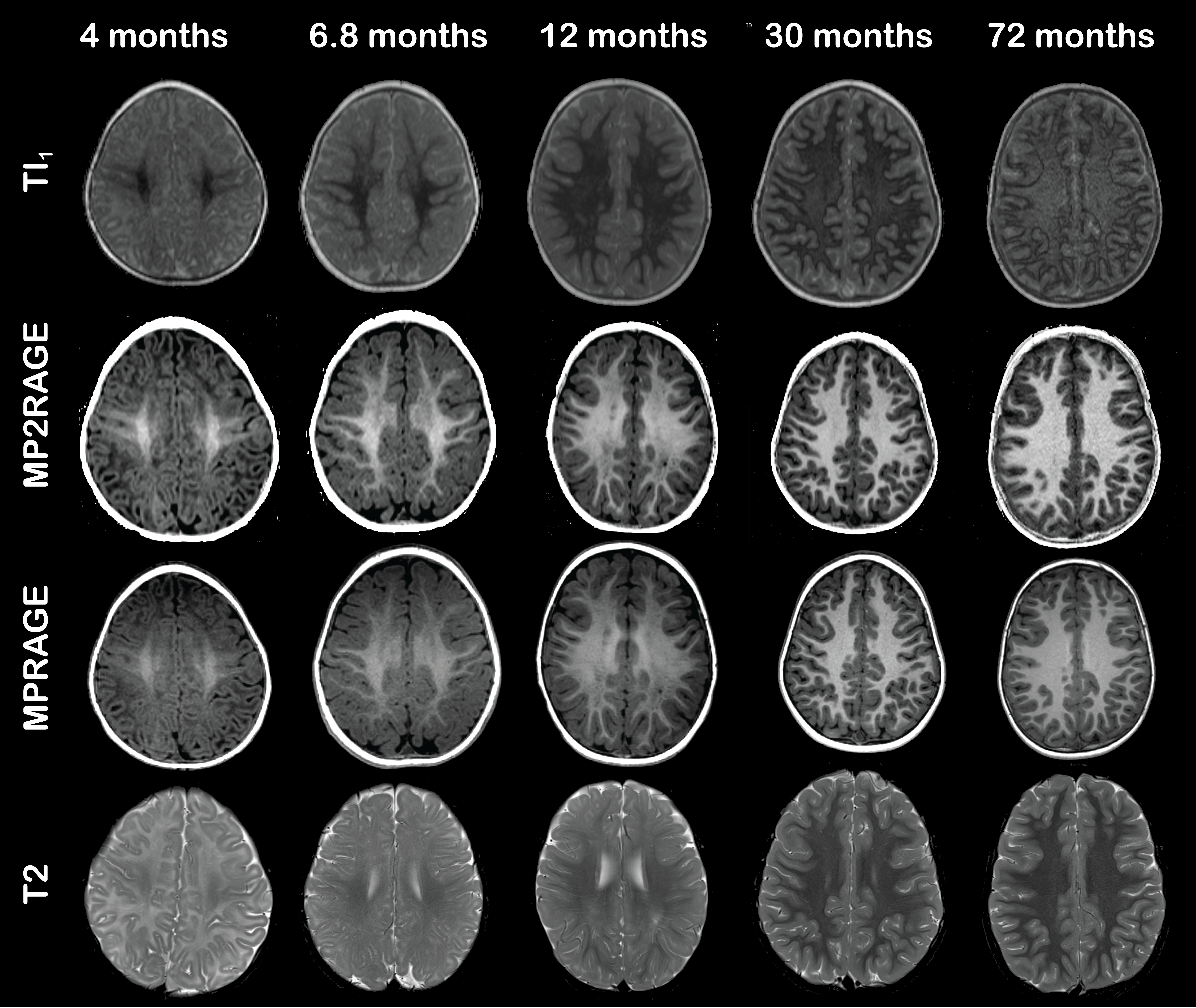

Supplement: Supplementary file 4 — Axial TI1, MP2RAGE, MP-RAGE and T2-weighted images at the level of the centrum semiovale in different age groups (boys ages 4 months, 6.8 months and 30 months, and girls ages 12 months and 72 months)(PNG 9.19 mb) [file 247_2021_5109_MOESM4_ESM.png]
